# Efficient Long Short-Term Memory-Based Sentiment Analysis of E-Commerce Reviews

**DOI:** 10.1155/2022/3464524

**Published:** 2022-06-17

**Authors:** Naveen Kumar Gondhi, Chaahat, Eishita Sharma, Amal H. Alharbi, Rohit Verma, Mohd Asif Shah

**Affiliations:** ^1^Shri Mata Vaishno Devi University, Katra, Jammu & Kashmir, India; ^2^Department of Computer Sciences, College of Computer and Information Sciences, Princess Nourah bint Abdulrahman University, P.O. Box 84428, Riyadh 11671, Saudi Arabia; ^3^School of Computing, National College of Ireland, Dublin, Ireland; ^4^Bakhtar University, Kabul, Afghanistan

## Abstract

In today's modern era, e-commerce is making headway through the process of bringing goods within everyone's grasp. Consumers are not even required to step out of the comfort of their homes for buying things, which makes it very convenient for them. Moreover, there is a wide variety of brands to choose from. Since more customers depend on online shopping platforms these days, the value of ratings is also growing. To buy these products, people rely solely on the reviews that are being provided about the products. To analyze these reviews, sentiment analysis needs to be performed, which can prove useful for both the buyers and the manufacturer. This paper describes the process of sentiment analysis and its requirements. In this paper, Amazon Review dataset 2018 has been used for carrying out our research and Long Short-Term Memory (LSTM) has been combined with word2vec representation, resulting in improving the overall performance. A gating mechanism was used by LSTM during the training process. The proposed LSTM model was evaluated on four performance measures: accuracy, precision, recall, and F1 score, and achieved overall higher results when compared with other baseline models.

## 1. Introduction

Communication has played a key role in boosting social relationships since historical times. Nowadays, nearly every segment of society uses social media as it has evolved into an efficacious networking tool. The main part of social media comprises e-commerce sites. Because of the rapid advancement of e-commerce technologies, the majority of people now choose to purchase online. People can use social media to provide feedback on various situations, items, and resources, which can be positive or negative, based on the customer's experience. Unfavorable comments play an essential role in the growth of the company because they help to improve the services. Here, sentiment analysis comes into play.

**Figure 1:**
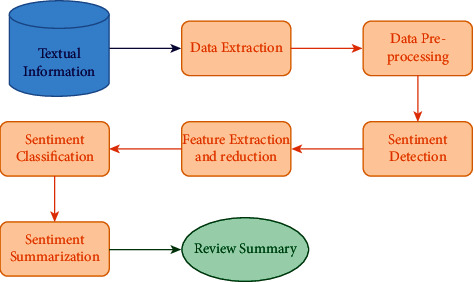
Stages of sentiment analysis.

Sentiment analysis aids in giving away a customer's viewpoint on different goods via text information and at the same time assessing these reviews shared. Various researches suggest that sentiment analysis is generally conducted at three levels: sentence, document, and phrase-level [1–3]. The substeps involved in the process of sentiment analysis are depicted in Figure 1.

This research proposes the use of LSTM networks to classify a large number of Amazon reviews. This deep learning technique is fast and gives better results even for a large number of reviews. The study uses word2vec embedding for the efficient estimation of word representations in vector space. Word2vec provides better results than the standard representation methods such as bag of words or one-part encoding. This study mainly focuses on two parts: Efficient mapping of sentiment words into vector space through the word2vec model and the LSTM network to classify reviews.

## 2. Literature Survey

This section contains all important background work on the subject of sentiment analysis that is relevant to our research. We discovered that most of the earlier works have employed machine learning algorithms, deep learning algorithms, and sentiment lexicon. In Table 1, we have summarized the approaches used in the research and the merits and demerits of the approaches.

In the year 2013, Sindhu and Chandrakala [4] observed recent and efficient techniques that are employed to study sentiment analysis, including sentiment polarity classification and various machine learning techniques such as Naive Bayes, Maximum Entropy, and support vector machine. The survey suggests that sentiment classification can be determined by two attributes, polarity assignment, i.e., determining if the sentiment is positive, negative, or neutral, and intensity assignment, which depicts how strong or mild a particular sentiment is in terms of polarity. Jurek et al. [5] presented a model with a lexicon-based sentiment analysis algorithm that included two key components: evidence-based integration function and sentiment normalization that measured emotion rather than a positive/negative label and aid in the differentiation of different emotions. A publicly available Twitter Corpus was used as a dataset for this study, the main focus of the study being real-time Twitter content analysis.

Zhang et al. [6] presented a multiclassification approach to perform sentiment analysis on e-commerce reviews. Further, Zhang et al. [6] presented a multiclassification model for sentiment analysis of e-commerce reviews. The Amazon review dataset (2018) was used for the proposed study, which was based on a directed weighted problem. The proposed study stated that, by extraction of entity words with features, assessment of sentiment patterns, and evaluation of the shortest path between nodes, the problem of sentiment similarity could be transformed into a problem of shortest path computation. When compared to the BERT model [7], this model performed better in terms of the algorithm's CPU time.

Dey et al. [8] examined the machine learning algorithms, K-NN and Naive Bayes, using three evaluation metrics. The Naive Bayes classifier outperformed the K-NN classifier in their work. Researchers in [9] presented a sentiment classification model with two techniques. The first proposed approach, the sentiment classification algorithm, employed the K-NN classifier and in the other one, the support vector machine algorithm was used. The efficiency of the classification algorithm was validated based on real tweets. The results obtained showed that the sentiment classification algorithm outperformed SVM on experimental validation. In [10], a comparison of supervised and unsupervised learning methods was presented. Their work provided a comparative analysis of supervised (CNN and KNN) and unsupervised (CNN with K means clustering) learning algorithms.

Fang et al. [11] introduced a multisentiment analysis technique that heavily incorporates fuzzy set theory, machine learning theory, and a polarity lexicon-based method. Consumer reviews were then analyzed using this hybrid model. Naive Bayes and SVM algorithms were used for this study. The enhanced SVM model, i.e., a hybrid method that combines multistrategy sentiment analysis with the SVM, was much more successful and gave an accuracy of 86.35%. Additionally, a 3.8% increase in accuracy was observed while implementing the upgraded Naive Bayes. In addition, researchers in [12] presented a way for incorporating lexical embeddings and an attention mechanism into CNN. The dataset was created using tweets. The method was evaluated using the F1 score. The work that was suggested performed better than the present ones.

A Recursive Neural Network (RNN) based recommendation system (RDSA) was introduced by Preethi et al. [13]. Deep learning was utilized to optimize suggestions centered on sentiment analysis and was done on three separate reviews in this study. Firstly, datasets were investigated and their statistical aspects were observed before implementing the Naive Bayes classifier and the RNN. The results of the trials showed that using RNN, a deep neural network, boosted the accuracy of sentiment analysis, leading to finer suggestions for users and aiding in the selection of a particular position depending on the requirements of the users.

Furthermore, researchers in [14] proposed using a Gini index-based feature selection and an SVM classifier to categorize data. The dataset for this study was a large collection of movie reviews. Based on the findings of the experiments, the proposed method was determined to be less accurate than other methods. A gated RNN with interopinion connections was introduced by Chen et al. [15]. This approach had an accuracy of about 92.6%. For classification, a bidirectional gated recurrent unit (BiGRU) paired with an attention mechanism was proposed in [16]. This approach was found to be effective for classification tasks and generated better outcomes than previously utilized methods, with a 93.1% accuracy. A replacement sentiment analysis model that incorporates the CNN and the attention-based BiGRU was proposed by researchers in [17]. By integrating the benefits of sentiment lexicon with deep learning technology, it compensates for flaws in the traditional sentiment analysis model for product reviews. The sentiment lexicon supports the sentiment attributes found in the reviews and CNN used in conjunction with the gated recurrent unit network extracts significant sentiment features and context elements. The suggested model gave 93.5% accuracy in the experimental analysis, which was found to be higher than the NB, SVM, and CNN models. Hyun et al. [18] suggested a convolutional neural network model based on target dependence. The recommended method helps in assessing the impact of the surrounding words on the target word by computing the distance between the target word and the surrounding words. Their study found that each term in a sentence had a varied effect on the statement's emotional polarity.

A hybrid deep learning model that systematically integrates multiple word embedding approaches (Word2vec, FastText, and character-level embedding) and several deep learning methods (LSTM, GRU, BiLSTM, and CNN) was proposed by researchers in [19]. The suggested model obtains features by extracting them by using various word embedding methods, merges them, and classifies text as per sentiment. To validate the suggested model's performance, numerous deep learning models known as standard models were built and used to run a series of experiments. When comparing the performance of the proposed model with that of earlier research, the new model outperforms the baseline models, according to the findings of this study.

Muhammad et al. [20] presented a model for sentiment analysis by using word2vec and LSTM for hotel reviews. For this study, the data was collected by crawling the travel website using selenium and scrap. The foremost purpose of this experiment was to analyze the accuracy by changing the parameters of word2vec and LSTM. The results showed that the mean accuracy of 85.96 could be achieved using the parameters, which showed promising results.

Zhao et al. [21] introduced a new technique to analyze the customers' sentiments from reviews on e-commerce websites. The proposed optimized technique “the Local Search Improvised Bat Algorithm based Elman Neural Network (LSIBA-ENN)” involves four steps and detects the polarity and classifies the sentiments of the reviews. The data for this research was gathered by using the web scrapping tool on e-commerce websites to extract customer reviews. In addition to preprocessing the data, this study utilizes “Log Term Frequency-based Modified Inverse Class Frequency (LTF-MICF) and Hybrid Mutation based Earth Warm Algorithm (HMEWA)” for term weighting and feature selection. The proposed methodology outsmarted other baseline techniques in terms of prediction accuracy.

Jiang [22] proposed a model to classify the sentiments of reviews obtained from the e-commerce platform Taobao. The study utilizes the machine learning algorithm as well as support vector machine for classification and improved particle swarm optimization (IPSO) to optimize the parameters. The data for the study was gathered by crawling the comments from the website. The experimental results demonstrated that the combined approach of SVM and IPSO had higher accuracy. However, the majority of the existing models suffer from overfitting [23–25], poor convergence speed [26–28], and vanishing gradient problems [29–31].

## 3. Experimental Study

This section gives a clear overview of the methodology used in the project for the classification of sentiment. The technique that has been used is a Long Short-Term Memory network, which is used to classify a large number of Amazon database reviews. The embedding used is word2vec, which has been custom trained according to the database. Tuning the word2vec according to the dataset improves the overall performance of the model. The benefit of using LSTM is that it gives better results even for the unstructured review data. It is capable of obtaining useful functionality for resources containing long-term dependencies.

**Figure 2:**
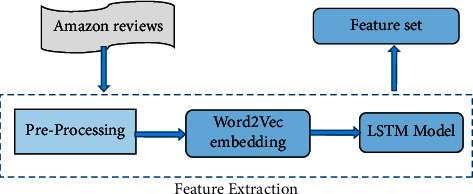
Process flow for obtaining features.

The data is collected from the Amazon review dataset, which is then preprocessed. Word2vec embeddings form an important step in the preprocessing of the data. Train and test data were created. The training data is split into train and validation datasets. The custom word2vec model is trained per database. The feature vector is obtained, which is then used as the embedding layer for the LSTM model. Keras is used for building the LSTM sequential model with max features equal to 50,000 and embedding size equal to 16. The model is then trained for 10 epochs. The model is tested based on sklearn performance metrics. The process of obtaining features is depicted in Figure 2.

### 3.1. Dataset

To generate accurate results, the dataset used should be large and enriched. The dataset has been collected from the cell phone and accessories section online of the Amazon Reviews dataset (2018). The dataset consists of a total of 938,261 reviews, among which 47901 are of unique products and 153124 are unique user reviews. The dataset initially consists of 7 columns, namely, a rating which varies from 1 to 5, review time, reviewer id, product id, and review text summary. After dropping the duplicates, the dataset consists of 938254 records, and Table 2 shows a snippet of the original dataset records.

### 3.2. Methodology

We have custom trained our word2vec model to be used with the LSTM model for classification. Word2vec is a word embedding which is used to represent a word by a collection of a number of terms of a vector. It is a way of mapping a word into a vector space. The dataset is loaded into a pandas data frame. For developing a custom word2vec model, the first step is preprocessing of data. We only look at the rating and review text and drop everything else. The text is cleaned by removing the punctuation. A subsample of the text is created from close to 200,000 reviews and the clean text method is applied to convert every review into a list of words. This list of words now acts as the input to the genism word2vec model.

We have built a custom trained skip-gram word2vec model and instantiated the model with dimensions: the size of word vectors as 100, window size equal to 15, min_count as 2 for words appearing less than 2 times in our corpus, negative equal to 5, and sampling rate equal to 1e−5. We have used all these dimensions to build a vocabulary from our review sentences.

We train our word2vec model for 1000 epochs. Then we compute the loss at every epoch. The loss is high at the beginning and it decreases towards the last epoch. The loss at epoch 0 is 2239394.0 and the loss at epoch 1000 is 11504.0. The saved model is then reloaded and operations are performed on it. For example, if we want to find words similar to noise in our dataset, we get canceling and headphones. Similarly, we can also find the similarity between certain words such as earphones and headphones which is 0.48756, and the similarity between the words charge and charger is 0.89264.

To reduce the dimensions of our data, we have used TSNE visualization to plot the data into two dimensions. Now, these word vectors can be used for further classification. These embeddings are then used as features for further streaming.

#### 3.2.1. Data Preparation for LSTM

Our dataset consists of 938254 records with most of the reviews having a score distribution of more than 3. We have first calculated the number of words for each review. The average mean is used as statistics to find the average length of reviews. The mean length of the review is 44.59 and the maximum length is 4303. We have created a dataset consisting of reviews having 100 words or less. Reviews whose length is more than 20 but less than 100 are categorized under short reviews and the rest are categorized under long reviews. The number of short reviews is 411313 and long reviews are 100239. Hyperparameters used in the model are described in Table 3.

Next, we have defined the sentiment rating as positive if the rating is greater than or equal to 3; else the rating is negative. We have considered the review text and sentiment for creating the train data set. The test data consists of products having at least more than 10 reviews. After distribution, the training dataset consisted of a total of 203891 records, among which 175910 belonged to the positive class and 27981 to the negative class. The test dataset consisted of a total of 686345 records, among which 592118 belonged to the positive and 94227 to the negative class.

**Figure 3:**
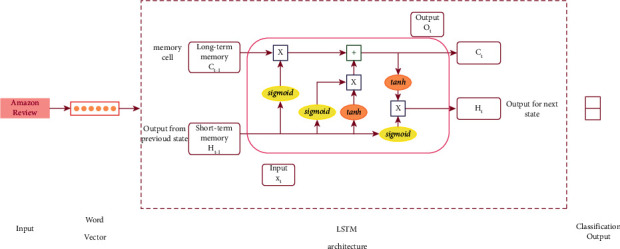
Proposed LSTM architecture.

In this study, we have used Keras to build our LSTM model, which takes a maximum of 50,000 features as the input to the embedding layer. Long short-term memory (LSTM) is a type of recurrent neural network which uses an internal mechanism that regulates the flow of information. This internal mechanism consists of gates that need to be trained such that they can accurately filter out irrelevant information and retain useful information.  Figure 3 shows the basic architecture of the LSTM model in our proposed methodology. *H*_*t*−1_ and *X*_*t*_ are the inputs to the LSTM unit; *H*_*t*−1_, commonly referred to as short-term memory, takes the output from the previous states as the input. The memory cell or the long-term memory, *C*_*t* −1_, helps in carrying relevant information throughout the process of a sequence. The LSTM architecture combines three gates: forget gate, input gate, and output gate. In the LSTM unit, tanh and sigmoid functions are used to obtain these gates.

The train data was then split into train and validation data of equal length. The length of the data was calculated to be 101945 and the class distribution was {1 : 87955, 0 : 13990}. To create the TensorFlow train test and validation datasets, we need to convert our train data into sequences. We have padded them to a maximum length of 100 so that all sequences are of the same length. The train and test labels are then converted to an NPS array. The TensorFlow dataset distribution is as follows:

  Train data: 101945, Class distribution {1 : 87955, 0 : 13990}
  Validation data: 101946, Class distribution {1 : 87955, 0 : 13991}
  Test data: 686345, Class distribution {1 : 592118, 0 : 94227}

**Figure 4:**
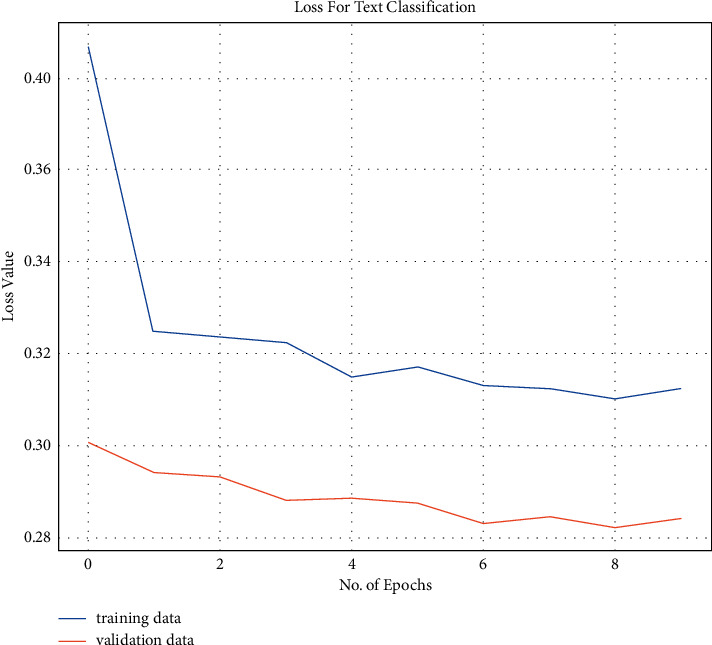
Training and validation loss.

All reviews are now present in the form of tensors of dimension 100. We have used Keras to build our model, a software library that acts as an interface for the TensorFlow library. It is integrated as a part of TensorFlow 2.1. For the final classification, we have used activation as a sigmoid due to the binary classification problem. The architecture of the model is shown in Figure 4. The language used for implementing the model is Python. When we compile the model, we use the Adam optimizer. The metric used for calculating loss is binary cross-entropy. For accuracy, the default threshold is taken as 0.5. If the output probability is above 0.5, it says the review belongs to the positive class, or else it belongs to the negative class. As we can see from the model architecture, the model takes the batch size as input followed by a length of 100. We train our model for about 10 epochs by creating batches of 1024 size as per the GPU available. The accuracy and loss of the model are calculated and plotted.

## 4. Results

**Figure 5:**
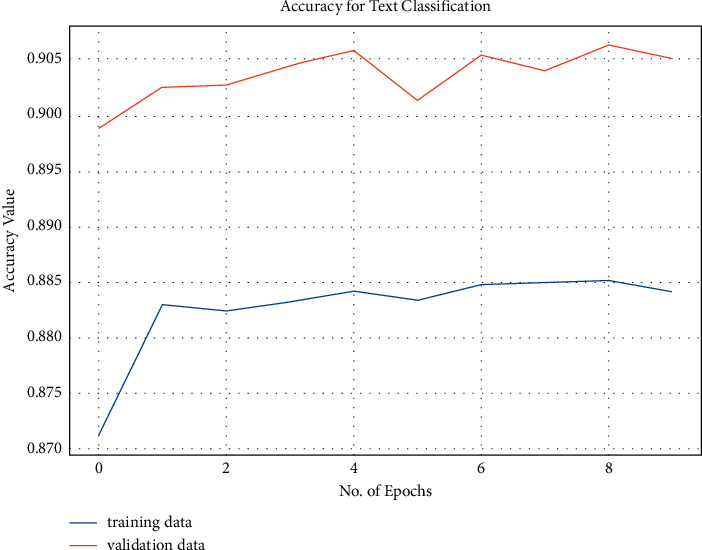
Training and validation accuracy.

We trained our model for about 10 epochs and calculated the training and validation loss as well as training and validation accuracy. We can see from Figure 4 that both the training and validation loss decreased throughout the training of the model.  Figure 5 shows that the training and validation accuracy increased subsequently for 10 epochs.

**Figure 6:**
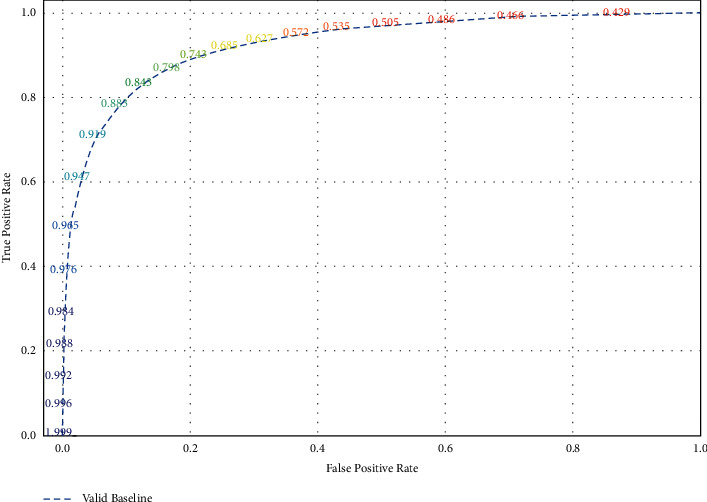
ROC curve.

Since, after prediction, the final output we get is a probability, we apply a certain threshold to determine whether the data belongs to the positive or negative class. For this purpose, we have used the ROC curve that plots the true positive rate and true negative rate. It helps find the threshold values for a binary classifier. From our ROC curve shown in Figure 6, we have chosen the value 0.78 as our threshold.

The saved model is reloaded and predictions have been generated on the test data considering the abovementioned threshold value. Now we have the original sentiment as well as the predicted sentiment. Since the dataset is imbalanced, the better parameter to test the model would be the F1 score rather than accuracy. In Table 4, we have compiled the accuracy, precision, recall, and F1 score of other baseline models and compared them with our model. The baseline models were considered from the literature we reviewed for this experiment.

## 5. Conclusion

This paper discusses sentiment analysis in the context of e-commerce reviews. There have been various techniques surveyed previously in the field of opinion mining of reviews. Our database consists of reviews from the cell phone and accessories section of Amazon. Long Short-Term Memory Networks were used to classify the sentiment using deep learning. Our custom training dataset was used to extract the features embedded in the word2vec embedding technique. Based on the ROC curve, we determined that 0.78 is the final threshold we should use to classify sentiment. Four parameters have been used to evaluate our model's performance: accuracy, precision, recall, and F1 score. A precision of 97% is found to be the highest of the four parameters. As the dataset is unbalanced, we consider the F1 score as the best measure of the performance of the model, which yields an evaluation of 93%. The main attempt of this research was to test the functionality of the model with a large amount of data. This method provides good results even for such large data of about 938,261 reviews. The main advantage of using this method is that LSTM takes into consideration long-term memory and word2vec efficient estimation of word representations which help in efficient sentiment analysis. For future work, we would like to consider using bidirectional LSTM for sentiment classification which trains two strands of LSTMs, the actual input sequence and the reverse one. This might help improve the model performance.

## Figures and Tables

**Table 1:** Comparative analysis of different approaches.

Author	Approaches	Advantages	Disadvantages
S. Zhang et al.	Sentiment multiclassification	Accuracy was higher than the other model	The model was found to have a high cost
L Dey et al.	Naive Bayes	Easy computation and better accuracy than KNN	Similar precision was observed in KNN and Naive Bayes for hotel review sets
M. R. Huq et al.	Support classification algorithm (SCA)	The accuracy of the model increases by normalizing the dataset	The model performs poorly for larger datasets
B. S. Lakshmi et al.	CNN	The models showed good results on both smaller and larger datasets	Better results are observed by combining the attention method
Y. Fang et al.	Enhanced NB, enhanced SVM	Feature values and sentiment values are combined	Only slightly higher accuracy than support vector machine and Naive Bayes
B. Shin et al.	CNN, attention	Attention mechanism helps reduce noise	The model does not consider multiple words
G. Preethi et al.	Naive Bayes and recursive neural network	Boosted the accuracy of the sentiment analysis system	This model only considers small datasets
A. S. Manek et al.	Feature selection using Gini index, support vector machine	The model works with both smaller and larger datasets	This method results in high cost
C. Chen et al.	BiGRU	This method effectively captures sentimental relations	—
L. Zhou and X. Bian	BiGRU, attention	The accuracy is improved by using the attention mechanism	—
L. Yang et al.	SLCABG	This method combines the benefits of both CNN and BiGRU in one model	The method proves to be of high cost without any sentiment multiclassification

**Table 2:** Snippets of the original data set.

Serial number	Rating	ReviewTime	ReviewerID	Product ID	Review text	Unix review time
0	5	08 4, 2014	A24E3SXTC6LJI	7508492919	Looks even better in person. Be careful not to…	1407110400
1	5	02 12, 2014	A269FLZCB4GIPV	7508492919	When you don't want to spend a whole lot of ca…	1392163200
2	3	02 8, 2014	AB6CHQWHZW4TV	7508492919	So the case came on time, I love the design. I…	1391817600
3	2	02 4, 2014	A1M117A53LEI8	7508492919	Don't care for it. gave it as a gift and they…	1391472000
4	4	02 3, 2014	A272DUT8M88ZS8	7508492919	I liked it because it was cute, but the studs…	1391385600
5	2	01 27, 2014	A1DW2L6XCC5TJS	7508492919	The product looked exactly like the picture an…	1390780800
6	3	01 23, 2014	AQC61R4UST7UH	7508492919	I Finally got my case today. It took forever t…	1390435200
7	5	01 17, 2014	A31OVFL91BKXG	7508492919	It is a very cute case. None of the jewels hav…	1389916800

**Table 3:** Hyperparameters used in the methodology.

Parameter	Value
Maximum length	100
Epochs	10
Optimizer	Adam
Loss function	Binary cross-entropy
Activation	Sigmoid
Dropout	0.4
Hidden activation	ReLU

**Table 4:** Performance evaluation comparison.

Approach used	Accuracy (%)	Precision	Recall	F1 score
Naive Bayes	57.9	55.6	79.2	65.3
Support classification algorithm (SCA)	67.7	93.5	38.4	54.5
CNN	90.9	91	90.2	90.6
LSTM	89	97	90	93

## Data Availability

The data used to support the findings of this study are available from the corresponding author upon request.

## References

[1] Liu B. (2015). *Sentiment Analysis: Mining Opinions, Sentiments, and Emotions*.

[2] Balaji P., Nagaraju O., Haritha D. March). Levels of sentiment analysis and its challenges: a literature review.

[3] Varghese R., Jayasree M. (2013). A survey on sentiment analysis and opinion mining. *International Journal of Renewable Energy Technology*.

[4] Sindhu C., Chandrakala D. S. (2013). A survey on opinion mining and sentiment polarity classification. *International Journal of Emerging Technology and Advanced Engineering*.

[5] Jurek A., Mulvenna, Bi Y. (2015). Improved lexicon-based sentiment analysis for social media analytics. *Security Informatics*.

[6] Zhang S., Zhang D., Zhong H., Wang G. (2020). A multiclassification model of sentiment for E-commerce reviews. *IEEE Access*.

[7] Devlin J., Chang M., Lee K., Toutanova K. BERT: pre-training of deep bidirectional transformers for language understanding.

[8] Dey L., Chakraborty S., Biswas A., Bose B., Tiwari S. (2016). Sentiment analysis of review datasets using naive Bayes and k-nn classifier. https://arxiv.org/abs/1610.09982.

[9] Huq M. R., Ali A., Rahman A. (2017). Sentiment analysis on Twitter data using KNN and SVM. *International Journal of Advanced Computer Science and Applications*.

[10] Lakshmi B. S., Raj P. S., Vikram R. R. (2017). Sentiment analysis using deep learning technique CNN with KMeans. *International Journal of Pure and Applied Mathematics*.

[11] Fang Y., Tan H., Zhang J. (2018). Multi-strategy sentiment analysis of consumer reviews based on semantic fuzziness. *IEEE Access*.

[12] Shin B., Lee T., Choi J. D. (2016). Lexicon integrated CNN models with attention for sentiment analysis. https://arxiv.org/abs/1610.06272.

[13] Preethi G., Krishna P. V., Obaidat M. S., Saritha V., Yenduri S. Application of deep learning to sentiment analysis for recommender system on cloud.

[14] Manek A. S., Shenoy P. D., Mohan M. C., Venugopal K. (2017). Aspect term extraction for sentiment analysis in large movie reviews using Gini Index feature selection method and SVM classifier. *World Wide Web*.

[15] Chen C., Zhuo R., Ren J. (2019). Gated recurrent neural network with sentimental relations for sentiment classification. *Information Sciences*.

[16] Zhou L., Bian X. (2019). Improved text sentiment classification method based on BiGRU-attention. *journal of physics: conference series*.

[17] Yang L., Li Y., Wang J., Sherratt R. S. (2020). Sentiment analysis for E-commerce product reviews in Chinese based on sentiment lexicon and deep learning. *IEEE Access*.

[18] Hyun D., Park C., Yang M.-C., Song I., Lee J.-T., Yu H. (Jul. 2019). Target-aware convolutional neural network for target-level sentiment analysis. *Information Sciences*.

[19] Salur M. U., Aydin I. (2020). A novel hybrid deep learning model for sentiment classification. *IEEE Access*.

[20] Muhammad P. F., Kusumaningrum R., Wibowo A. (2021). Sentiment analysis using Word2vec and long short-term memory (LSTM) for Indonesian hotel reviews. *Procedia Computer Science*.

[21] Zhao H., Liu Z., Yao X., Yang Q. (2021). A machine learning-based sentiment analysis of online product reviews with a novel term weighting and feature selection approach. *Information Processing & Management*.

[22] Jiang X. (2022). A sentiment classification model of E-commerce user comments based on improved particle swarm optimization algorithm and support vector machines. *Scientific Programming*.

[23] Jie D., Zheng G., Zhang Y., Ding X., Wang L. (2021). Spectral kurtosis based on evolutionary digital filter in the application of rolling element bearing fault diagnosis. *International Journal of Hydromechatronics*.

[24] Singh D., Kumar V., Kaur M., Jabarulla M. Y., Lee H.-No (2021). Screening of COVID-19 suspected subjects using multi-crossover genetic algorithm based dense convolutional neural network. *IEEE Access*.

[25] Xu Y., Li Y., Li C. (2021). Electric window regulator based on intelligent control. *J. Artif. Intell. Technol.*.

[26] Hahn T. V., Mechefske C. K. (2021). Self-supervised learning for tool wear monitoring with a disentangled-variational-autoencoder. *International Journal of Hydromechatronics*.

[27] Kaushik H., Singh D., Kaur M., Alshazly H., Zaguia A., Hamam H. (2021). Diabetic retinopathy diagnosis from fundus images using stacked generalization of deep models. *IEEE Access*.

[28] Mondal S. C., Marquez P. L. C., Tokhi M. O. (2021). Analysis of mechanical adhesion climbing robot design for wind tower inspection. *Journal of Artificial Intelligence Technology*.

[29] Balakrishna A., Mishra P. K. (2021). Modelling and analysis of static and modal responses of leaf spring used in automobiles. *International Journal of Hydromechatronics*.

[30] Kaur M., Singh D., Kumar V., Gupta B. B., Abd El-Latif A. A. (2021). Secure and energy efficient-based E-health care framework for green internet of things. *IEEE Transactions on Green Communications and Networking*.

[31] Singh P. K. (2022). Data with non-Euclidean geometry and its characterization. *Journal of Artificial Intelligence Technology*.

